# Design and introduction of quaternary ammonium hydroxide‐functionalized graphene oxide quantum dots as a pseudo-homogeneous catalyst for epoxidation of α,β-unsaturated ketones

**DOI:** 10.1038/s41598-023-34635-5

**Published:** 2023-05-19

**Authors:** Mohammed Salim Mohammed, Homa Targhan, Kiumars Bahrami

**Affiliations:** 1 Nanoscience and Nanotechnology Research Center (NNRC), Razi University, Kermanshah, 67144-14971 Iran; 2 Department of Organic Chemistry, Faculty of Chemistry, Razi University, Kermanshah, 67144-14971 Iran

**Keywords:** Chemistry, Materials science

## Abstract

In present work, design and synthesis of a novel pseudo-homogeneous catalyst is described. For this purpose, amine-functionalized graphene oxide quantum dots (N-GOQDs) were prepared from graphene oxide (GO) by a facile one-step oxidative fragmentation approach. The prepared N-GOQDs were then modified with quaternary ammonium hydroxide groups. Various characterization techniques clearly revealed that the quaternary ammonium hydroxide‐functionalized GOQDs (N-GOQDs/OH^−^) have been successfully synthesized. TEM image revealed that the GOQDs particles are almost regularly spherical in shape and mono-dispersed with particle sizes < 10 nm. The efficiency of the synthesized N-GOQDs/OH^−^ as a pseudo-homogeneous catalyst in epoxidation of α,β-unsaturated ketones in the presence of aqueous H_2_O_2_ as an oxidant at room temperature was investigated. The corresponding epoxide products were obtained in good to high yields. This procedure has the advantages of a green oxidant, high yields, involvement of non-toxic reagents and reusability of the catalyst without discernible loss in activity.

## Introduction

In an era of new technologies, a safe and healthy living environment has become of great importance; as a result, the availability of more environmentally friendly chemical approaches for organic compounds production would also constitute an added advantage. The last two centuries have witnessed significant advances in the development of cost effective catalysts for organic transformation focusing on the some aspects of green chemistry^1,2^.

A pseudo-homogeneous catalyst has been considered as a catalytic system that the catalyst surface is exactly exposed to the substrates i.e. that there is no obvious phase differentiation between substrates and the catalyst and performs like a homogeneous catalyst. However, compared with homogeneous catalysts, the pseudo-homogeneous catalyst could be easily separated from reaction medium and recovered as is characteristic in heterogeneous catalysis. In turn, performance and usability of the catalytic system would be improved since the pseudo-homogeneous system will have the advantages of both the homogeneous and heterogeneous catalysis^3,4^.

Graphene oxide quantum dots (GOQDs), an emerging category of zero-dimensional nanomaterials, are defined as those oxygen-rich carbonaceous layered materials with constituent dimensions less than 20 nm^5^. GOQDs have shown promising potentials in electrochemical sensing, photocatalysis, bioimaging, biosensing, light-emitting diodes, and catalysis due to their exceptional properties such as remarkable luminescence, existence cost-effective methods to produce, easy functionalizing ability, very good solubility and stability in water, low toxicity and good biocompatibility^6–9^. It is interesting to note that GOQDs can be produced efficiently from various commercially available carbon sources including graphite powder, graphene oxide sheets, carbon fibers, citric acid, plant materials, such as mango leaves and so forth^6^.

Use of GOQDs as a support for active sites can be surprising since these catalytic systems allow the catalytic reaction occur under pseudo‐homogeneous conditions. Therefore, the catalytically active species can be suspended indefinitely due to the small particle size of CQDs and designed functional groups on it, and catalyst and reactants are in a same phase, so the system can work similarly to a homogeneous catalyst with the added advantage of being recoverable effortlessly by dialysis membrane. Furthermore, the thin sheet of GOQDs contain a range of reactive oxygen functional groups on their surface, which provides high (aqueous) solubility and considerable potential for facile modification^10–12^. On the whole, surface modification techniques can offer exciting possibilities for change the surface of GOQDs for particular applications^13^. Recently, GOQDs supported catalysts have been explored in organic transformations and they demonstrated excellent results. Rezaei et al. were able to perform the selective oxidative cracking of alkenes to aldehydes using the carbon quantum dots supported ionic liquids^14^. Immobilized tungstate ions on the surface of carbon quantum dots have been successfully applied in oxidative scission of alkenes and selective oxidation of alcohols to corresponding aldehydes^15–17^. Pd and Ag nanoparticles have also stabilized on carbon quantum dots and the prepared catalyst was utilized as an efficient catalyst for promoting the Suzuki–Miyaura coupling reaction^18^.

Epoxides have emerged as an extremely useful class of organic compounds with high synthetic utility. In organic chemistry, epoxides are useful and versatile synthon so that can be converted into a wide variety of valuable compounds with good pharmaceutical profiles^19^. Epoxides compounds are also a broad family of monomers for production of various types of polymers. A number of epoxides derivatives have been reported to show anticancer, antibiotic and protease inhibition activities^20–23^. Therefore, academic researchers have been giving much concentration to preparation epoxides-containing compounds.

Epoxidation of carbon–carbon double bonds is one of the most fundamental reactions. This transformation has a wide scope of applications in synthetic organic chemistry^24,25^. Despite the large number of undesirable environmental and economic effects continue to use stoichiometric oxidations such as sodium peroxide, sodium hypochlorite, m-chloroperbenzoicacid, oxone, sodium chlorite, sodium perborate tetrahydrate, dimethyldioxirane, cyclohexylidenebishydroperoxide and trichloroisocyanuric acid^26–34^. During the recent decade, catalytic protocols based on aqueous hydrogen peroxide (H_2_O_2_), air and pure oxygen as environmentally friendly, low-cost and readily available oxidants, are being considered^35^. Epoxidation of α,β-unsaturated ketones occurs in the presence of hydrogen peroxide under basic conditions and produces epoxy carbonyl compounds^36^. Representative examples of such systems are ([C_4_MIm][PF_6_]) ionic liquid/H_2_O_2_^37^, [Al(H_2_O)_6_]^3+^/H_2_O_2_^38^, (CTP)_3_VMo_12_O_40_/H_2_O_2_^39^, tetrabutylammonium peroxydisulfate/H_2_O_2_^40^, [L-Aaemim]Br ionic liquid/H_2_O_2_^41^, poly(L-Leucine)/H_2_O_2_^42^ and primary amine salts/H_2_O_2_^43^.

Although, great strides have been made in the development of affordable catalysts for the epoxidation of α,β-unsaturated ketones approach focusing on the principles of green chemistry, however, it continues to be one of the most interesting areas in scientific literature.

As part of our continuing investigations to develop green catalysts for organic transformations and with the aim of further demonstrating the catalytic potential of GOQDs, on the first step of this study, N-GOQDs were prepared from graphene oxide by an oxidative fragmentation approach. Subsequently, the prepared N-GOQDs modified with quaternary ammonium hydroxide groups. The catalytic activity of this pseudo-homogeneous catalyst (N-GOQDs/OH^-^) was evaluated in the epoxidation of α,β-unsaturated ketones in the presence of aqueous H_2_O_2_ as an oxidant at room temperature.

## Experimental

### Materials and apparatus

Used solvents and chemicals were supplied from Fluka (Switzerland) or Merck (Germany), and used without any further purification. Deionized (DI) water was applied in all tests. The Fourier-Transform Infrared Spectroscopy (FT-IR) spectra of the samples were recorded with the KBr pellet method by PerkinElmer PE-1600-FTIR spectrometer. Transmission electron microscopy (TEM) was investigated on an EM 208S (PHILIPS) 100 kV microscope with tungsten filament and Zeiss‐EM10C (Germany) operating at 100 kV with samples on formvar carbon coated grid Cu mesh 300. The prepared samples were investigated by field emission scanning electron microscope (FESEM) (FESEM TESCAN MIRA 3, Czech). The ^1^H NMR analysis was carried out with a BRUKER DRX-250 AVANCE spectrometer at 250.0 MHz. The optical characteristics of samples were measured by Shimadzu UV 2100 151PC UV–visible spectrophotometer at room temperature. Energy-dispersive X-ray spectroscopy (EDX) analysis were carried out on a SIGMA VP 500 (Zeiss) microscope equipped with an EDX measurement system.

### Synthesis of graphene oxide

First, the graphene oxide sheets have been prepared by the predominant modified Hummer’s method. Briefly, concentrated H_2_SO_4_ (15 mL) was added to a mixture of graphite (0.3 g) and NaNO_3_ (0.3 g), and the mixture was cooled to 0 °C in an ice‐salt bath. Under stirring, KMnO_4_ (1.5 g) was added slowly to the suspension over 2 h at 0–10 °C with ice‐salt bath cooling. The mixture was warmed to 35 °C and stirred for 30 min, and the resulting solution was diluted by slowly adding 30 mL of water under stirring. Then the reaction was stirred under reflux for 15 min at 98 °C. After cooling to room temperature, the resulting mixture was treated with 30% H_2_O_2_ solution (7 mL). The mixture was washed with HCl and H_2_O respectively, followed by centrifugation and drying, graphene oxide was thus obtained (0.35 g)^44^.

### Synthesis of N-GOQDs

0.02 g of as-prepared GO was dispersed in water (5 mL) under sonication conditions. *N*-(aminomethyl)methanediamine (1 mL) and H_2_O_2_ (40 mL, 30% solution in H_2_O). The mixture was then transferred into a single-neck, flat-bottom round flask and heated at 80 °C for 8 h. The product was then cooled to room temperature and was centrifuged to remove the largest particles and untreated GO sheets. Ultimately, the dark yellow solution was dialyzed using a dialysis membrane (100 Da) to remove the unreacted starting materials, then concentrated under vacuum, giving final volume of 5 mL^45^.

### Synthesis of N-GOQDs/OH^−^

5 mL methanol and 0.2 mL methyl iodide were added to 5 mL of N-GOQDs solution was. The mixture was stirred under reflux at 25 °C for 24 h. The product was then dialyzed using a dialysis membrane (100 Da) to remove the unreacted methyl iodide. Ultimately, 5 mL of quaternary ammonium iodide‐functionalized N-GOQDs (N-GOQDs/I) was treated with 3 mL KOH (0.1 M) at room temperature for 2 h to obtain quaternary ammonium hydroxide‐functionalized GOQDs (N-GOQDs/OH^−^)^46^. Finally, the resulting solution was dialyzed using a dialysis membrane (100 Da) to remove any excess KOH, then was concentrated under vacuum to a final volume of 5 mL.

### General procedure for epoxidation of α,β-unsaturated ketones using N-GOQDs/OH^−^

The catalysis experiments for the N-GOQDs/OH^−^ was carried out in a 1,4-dioxane:water (1:1) mixture, using 1:3 α,β-unsaturated ketones:H_2_O_2_ mole ratio. The α,β-unsaturated ketone (1 mmol) was introduced into a round‐bottom flask equipped, to which N-GOQDs/OH^−^ (4 mL of aqueous solution) and the mixture solvent (5 mL) were subsequently added. Then, aqueous 30% H_2_O_2_ solution (3 mmol) was added, the reaction solution was stirred (at 1000 rpm) at ambient temperature for 24 h. After detecting the completion of the reaction using thin-layer chromatography (TLC) (*n*-hexane/ethyl acetate, 2/1), the reaction mixture was diluted with water (5 mL), and the product was extracted with ethyl acetate (3 × 5 mL). The extracted catalyst in the aqueous layer was concentrated under vacuum to a final volume of 4 mL to reuse in another run. On the other hand, the organic phase (ethyl acetate layer) containing unreacted α,β-unsaturated ketones and the corresponding epoxide products were evaporated and purified by employing a silica gel column chromatography (ethyl acetate/*n*-hexane) to obtain the desired products.

## Results and discussion

### Synthesis and characterization of the catalyst

GO has been synthesized using the modified Hummer’s method. Hummer’s method is an efficient and reliable method for producing GO through the oxidation of graphite powder with potassium permanganate in the presence of sodium nitrate in a solution of sulfuric acid^47^. Thereafter GO has been transformed into N-GOQDs by a “top-down” oxidative fragmentation route in the presence of aqueous H_2_O_2_ and *N*-(aminomethyl)methanediamine (Supplementary Fig. S1). Finally, N-GOQDs/OH^−^ has been prepared by converting the amine groups to quaternary methyl ammonium iodide followed by ion-exchange with aqueous KOH solution, as shown in Fig. 2. The presence of various functional groups on the surface of the N-GOQDs/OH^−^ imparts high solubility in water and polar organic solvents, therefore, it can act as a pseudo-homogeneous catalyst.

The as-synthesized GOQDs was characterized using Fourier transform infrared spectroscopy (FTIR), Energy-dispersive X-ray spectroscopy (EDX), Transmission electron microscopy (TEM), Scanning electron microscopy (SEM), X-ray photoelectron spectroscopy (XPS) and Fluorescence spectroscopy.

**Figure 1:**
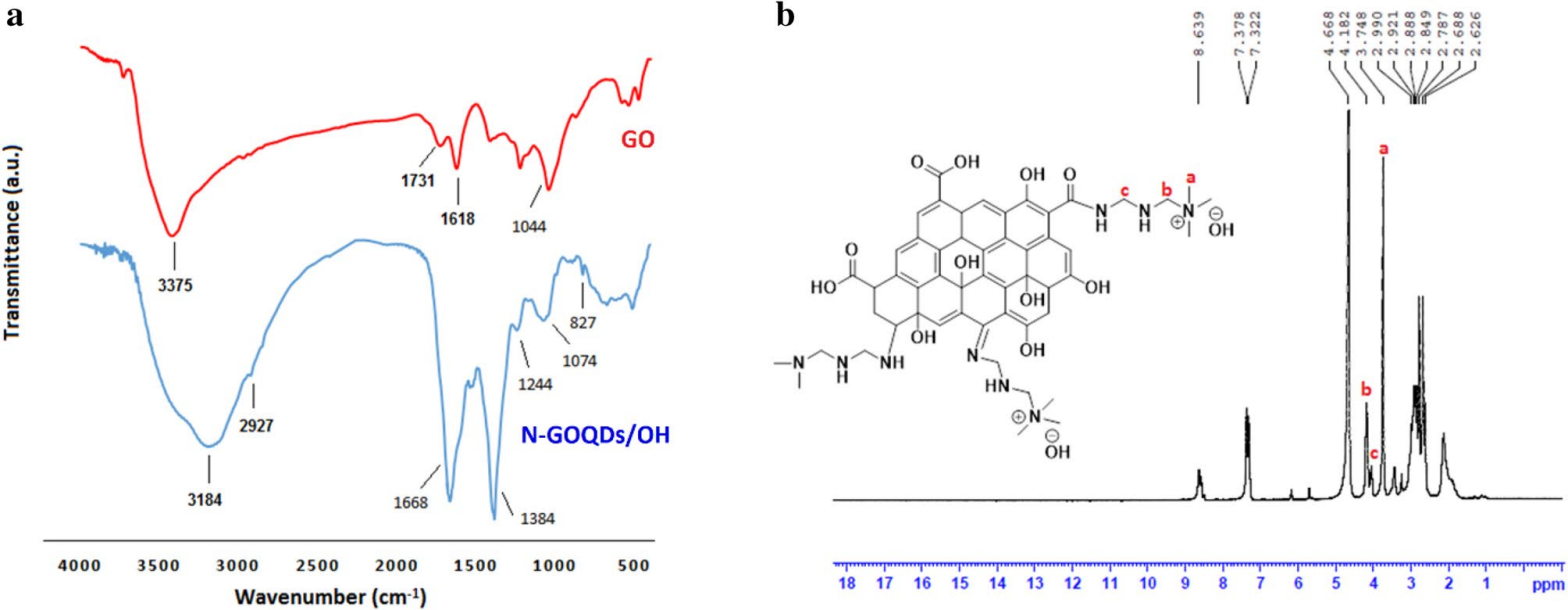
(**a**) FT-IR spectra of the GO and N-GOQDs/OH^−^ (**b**) ^1^H NMR spectrum of the N-GOQDs/OH^−^.

Figure 1a shows FT-IR spectra of the prepared GO and N-GOQDs/OH^−^. GO shows its characteristic peaks at 3375, 1731, 1618, and 1044 cm^−1^ arising due to O–H, C=O, C=C and C–O–C of the epoxides, respectively. After the oxidative fragmentation, the carbonyl and epoxide peaks disappear (1731 and 1044 cm^−1^ respectively)^45^. The presence of –OH and –NH group in N-GOQDs/OH^−^ was confirmed by the appearance of broad peak centered between 3000 cm^−1^ and 3500 cm^−1^. The strong signal at 1668 cm^−1^ can be correspond to the stretching vibration of C=N and C=O bonds, and the peak at 1384 cm^−1^ arise due to stretching vibration of C and N bond^48^. The presence of aliphatic C–H group is confirmed by the peak at 2927 cm^−1^. Therefore, the FT-IR image indicates that the compounds that contain nitrogen atoms are modified on the surface of the GOQDs. In the ^1^H NMR spectrum, dispersed in D_2_O, the presence of quaternary ammonium hydroxide groups could be identified (Fig. 1b).

**Figure 2:**
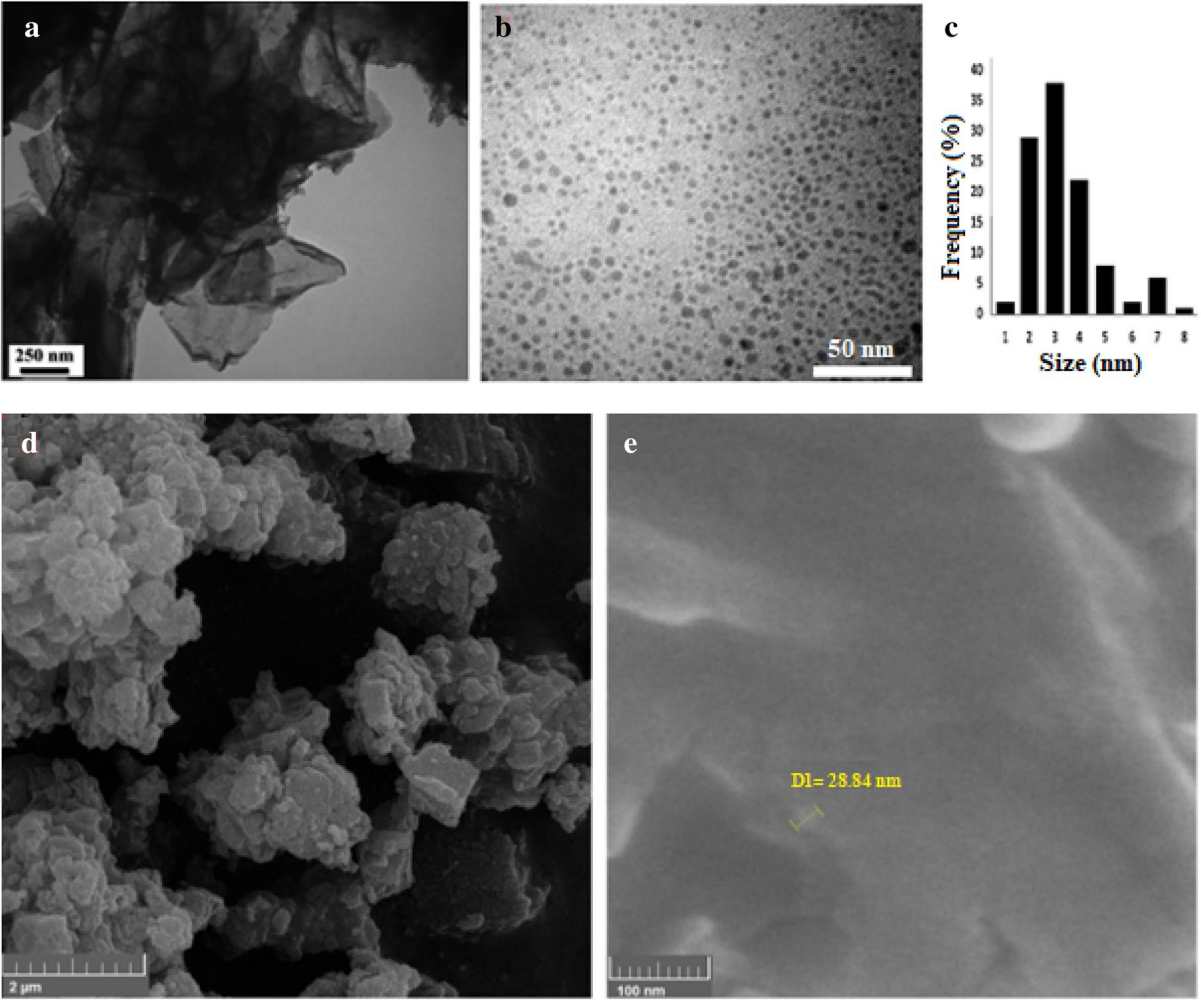
The TEM images of (**a**) the obtained GO, (**b**) N-GOQDs/OH^−^, and (**c**) particle size distribution, and FE-SEM image of (**d**) the obtained GO, (**e**) N-GOQDs/OH^−^.

Figure 2a,b shows TEM images of the obtained GO and the N-GOQDs/OH^−^ and Fig. 2c shows the particle size distribution. The average the N-GOQDs/OH^−^ size must be significantly smaller than the GO. The TEM images (Fig. 2b) clearly reveal the N-GOQDs/OH^−^ are nearly spherical in shape and mono-dispersed with particle sizes smaller than 10 nm whose mean size is very smaller than GO (Fig. 2a). Figures 2d,e show SEM images of the prepared GO and N-GOQDs/OH^−^, respectively. According to the FE-SEM image (Fig. 2e) of the GOQDs show sphere-shaped morphology.

To study the distribution and chemical composition of the N-GOQDs/OH^−^, EDX analysis was performed (Supplementary Fig. S2). A SEM image of the same region as the EDX is shown in Supplementary Fig. S2a. The elemental peaks attributed to carbon, oxygen, and nitrogen in the EDX spectrum confirmed the presence of these elements in the obtained N-GOQDs/OH^−^ (Supplementary Fig. S2b). Additionally, the EDX spectrum reveals the presence of an ignorable amount of potassium and calcium impurities (0.05 and 0.10 atom percent, respectively) (Supplementary Table S1). The presence of potassium can be related to an extremely small amount of KOH not removed in the dialysis step and the presence of calcium is a sign of environmental impurity in the catalyst identification stage. Therefore, the EDX spectrum confirmed good anchoring and stability of compounds that contain nitrogen atoms on GOQDs. Additionally, it can be inferred from the mapping images that elements of carbon, oxygen, and nitrogen are widespread in the area of N-GOQDs/OH^−^ because of the uniform formation of N-GOQDs/OH^−^ (Supplementary Fig. S2c).

**Figure 3:**
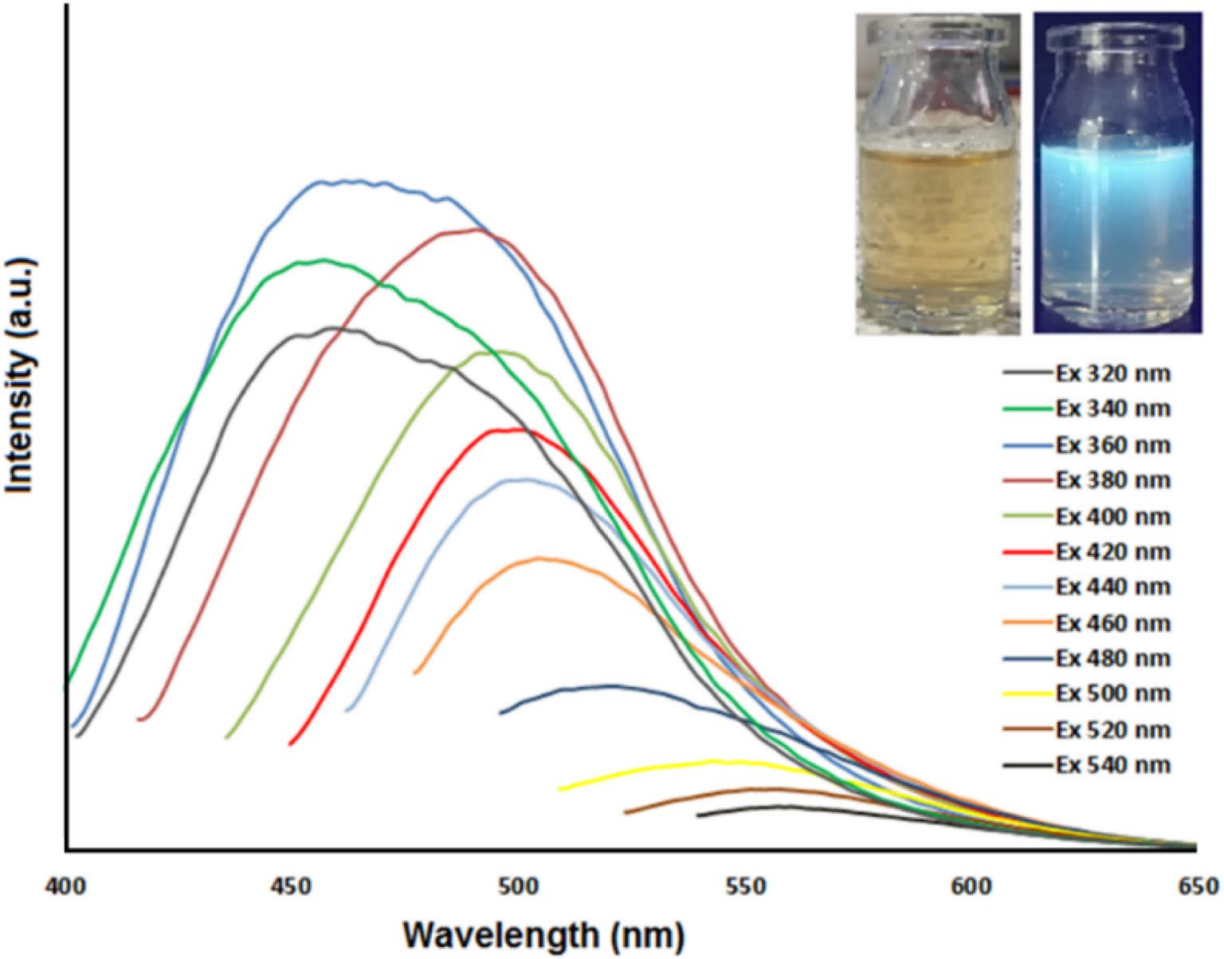
The fluorescence spectrum of the N-GOQDs/OH^−^.

Fluorescence spectroscopy is a simple and reliable methodology to confirm the quantum confined properties of semiconductor QDs^45,49^. The fluorescence response of the N-GOQDs/OH^−^ with regard to a variety of excitation wavelengths between 320 and 540 nm is displayed in Fig. 3. Consistent with previous research, the fluorescence intensity of the N-GOQDs/OH^−^ sample first increased and then decreased (Fig. 3). The samples displayed exhibited the strongest fluorescence peak with maximum emission centered at 445 nm at an excitation wavelength of 360 nm. The results clearly indicated the successful synthesis of the N-GOQDs/OH^−^ samples. Furthermore, photographs of an aqueous dispersion of N-GOQDs/OH^−^ under room light (left) and under 365 nm UV irradiation (right) shown in the figure.

**Figure 4:**
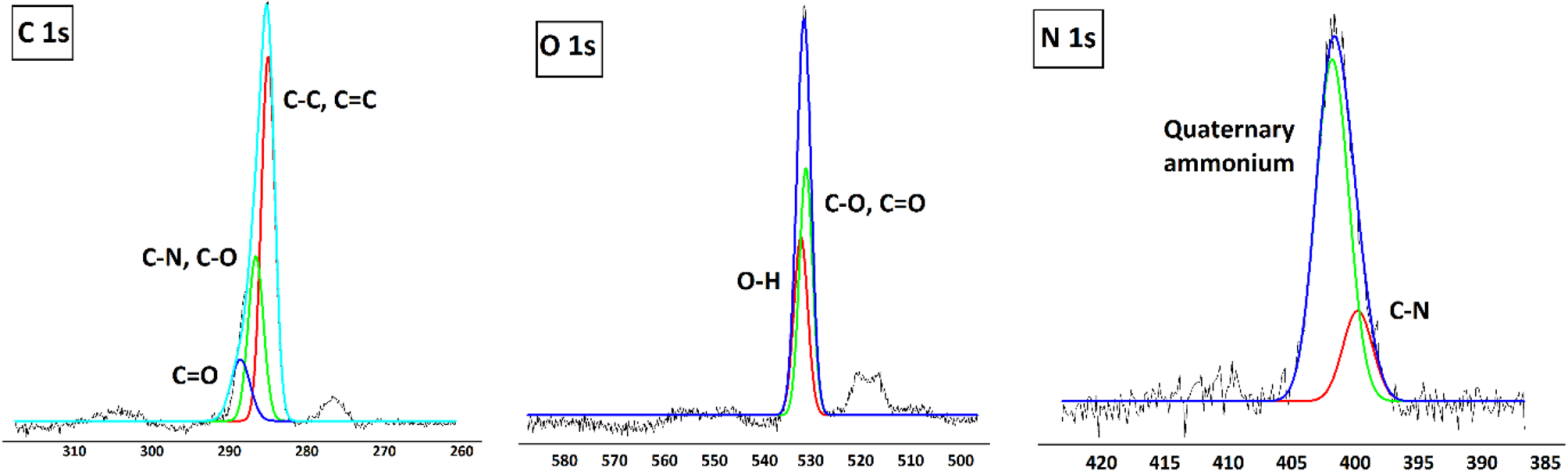
High resolution XPS spectra of C1s, O1s, N1s.

The atomic chemical states of synthesized N-GOQDs/OH^−^ sample was studied by XPS (Fig. 4). The full XPS spectra of N-GOQDs/OH^−^ clearly reveals the existence of carbon, oxygen and nitrogen (Supplementary Fig. S3). C1s spectrum shows peaks at BEs of 284 eV (referring to C–C and C=C), 286 eV (referring to C–O and C–N) and 288 eV (referring to C=O) (Fig. 4). The analysis of O1s shows peaks at BEs of 530 eV (assigned to C–O and C=O), and 532 eV (assigned to O–H) (Fig. 4). The N1s shows peaks at 400 eV and 402 eV corresponding to C–N and the quaternary ammonium group, respectively (Fig. 4)^15,45,50^.

### Investigation of catalytic activity of N-GOQDs/OH^−^ in epoxidation of α,β-unsaturated ketones

The catalytic activity of N-GOQDs/OH^−^, as a pseudo-homogeneous catalyst, was evaluated in epoxidation of α,β-unsaturated ketones. Initially, the epoxidation of benzalacetophenone was studied as standard substrate with the prepared nanocatalyst (Table 1). At the outset, many experimental parameters, such as catalyst amount, the H_2_O_2_/α,β-unsaturated ketones molar ratios and solvent have been studied in order to determine the optimal experimental conditions. The effect of reaction conditions on epoxidation of benzalacetophenone (1 mmol) with N-GOQDs/OH^−^ summarized in Table 1. As can be seen from the results in Table 1, in the absence of the catalyst, and in the presence of GO or N-GOQDs, no product obtained (Table 1, Entries 1–3) and an increase in the amount of the catalyst improve the yields significantly (Table 2, Entries 4–6). The effect of addition of H_2_O_2_ amount on the reaction was also studied. The yield significant improvements of epoxide product were observed with increasing amount of H_2_O_2_ (Entries 6–10). Investigating the role of solvent show that the protic solvents reduces the epoxide products yields (Entries 11–13). After careful examinations, the best result was obtained by using 4 mL of aqueous solution of catalyst, and in the presence of H_2_O_2_ (3 mmol) in 1,4-dioxane at ambient temperature (Table 1, Entry 6).

**Table 1:** The effect of reaction condition on epoxidation of benzalacetophenone with N-GOQDs/OH^−^.

Entry	Catalyst (mL of aqueous solution)	H_2_O_2_ (mmol)	Solvent	Time (h)	Yield (%)
1	–	3	1,4-Dioxane	24	**–**
2	GO (4)	3	1,4-Dioxane	24	**–**
3	N-GOQDs (4)	3	1,4-Dioxane	24	**–**
4	N-GOQDs/OH^−^ (2)	3	1,4-Dioxane	24	30
5	N-GOQDs/OH^−^ (3)	3	1,4-Dioxane	24	50
6	N-GOQDs/OH^−^ (4)	3	1,4-Dioxane	24	75
7	N-GOQDs/OH^−^ (4)	–	1,4-Dioxane	24	**–**
8	N-GOQDs/OH^−^ (4)	1	1,4-Dioxane	24	20
9	N-GOQDs/OH^−^ (4)	2	1,4-Dioxane	24	45
10	N-GOQDs/OH^−^ (4)	4	1,4-Dioxane	24	70
11	N-GOQDs/OH^−^ (4)	3	Methanol	24	65
12	N-GOQDs/OH^−^ (4)	3	H_2_O	24	–
13	N-GOQDs/OH^−^ (4)	3	EtOH–H_2_O	24	50
14	N-GOQDs/OH^−^ (4)	3	1,4-Dioxane	12	50

**Table 2:** Epoxidation of α,β-unsaturated ketones with N-GOQDs/OH^−^, ^a^All reactions were performed in the presence of N-GOQDs/OH^−^ (4 mL of aqueous solution), H_2_O_2_ (3 mmol) at room temperature for 24 h in 1,4-dioxane as solvent.

Entry^a^	Product	Conversion (%)	Selectivity (%)	M.P (°C) (lit.)
1		75	> 99%	80–84 (84–85)^51^
2		70	> 99%	116–120 (116–120)^51^
3		70	> 99%	54–57 (55–58)^41^
4		65	> 99%	110–116 (114–116)^52^
5		65	> 99%	60–70 (68–71)^41,51^
6		70	> 99%	60–69 (67–70)^51^
7		65	> 99%	57–60 (56–59)^51^
8		65	> 99%	140 (131–135)^51^
9		70	> 99%	136 (128–133)^50^
10		65	> 99%	144 (145–148)^50^

Under the optimized conditions, in a simple experimental procedure the transformations of a series of α,β-unsaturated ketones into the corresponding epoxides occurred in almost high yields. The results are summarized in Table 2. The present procedure is general as a wide range of α,β-unsaturated ketones derivatives with electron-donating and electron-withdrawing groups reacted easily with H_2_O_2_ at room temperature to afford the corresponding α,β-epoxy ketones in good to high yields and electronic effect of the substituents was not observed.

**Figure 5:**
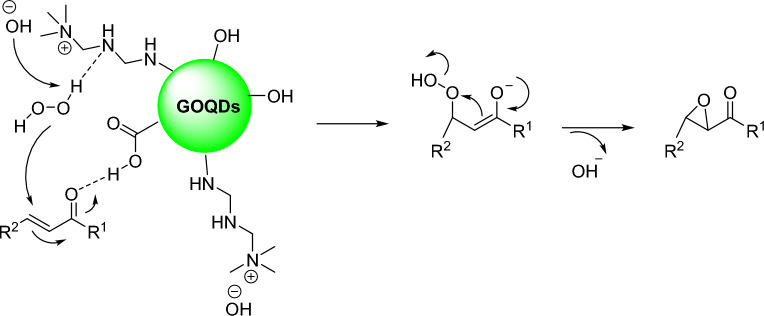
The plausible mechanism for the epoxidation reaction.

According to the literature, plausible mechanisms for the epoxidation of α,β-unsaturated ketones is proposed as shown in Fig. 5. The OH^−^ plays an essential role through formed HOO^−^ specie. On the other hand, the N-GOQDs/OH^−^ activate the α,β-unsaturated ketones through hydrogen-bond interaction. The reaction is followed by the nucleophile attack of the HOO^−^ and form a hydroperoxide enolate. Finally, the reaction is completed by lost the OH^-^ and formed the epoxide ring^53–56^.

**Figure 6:**
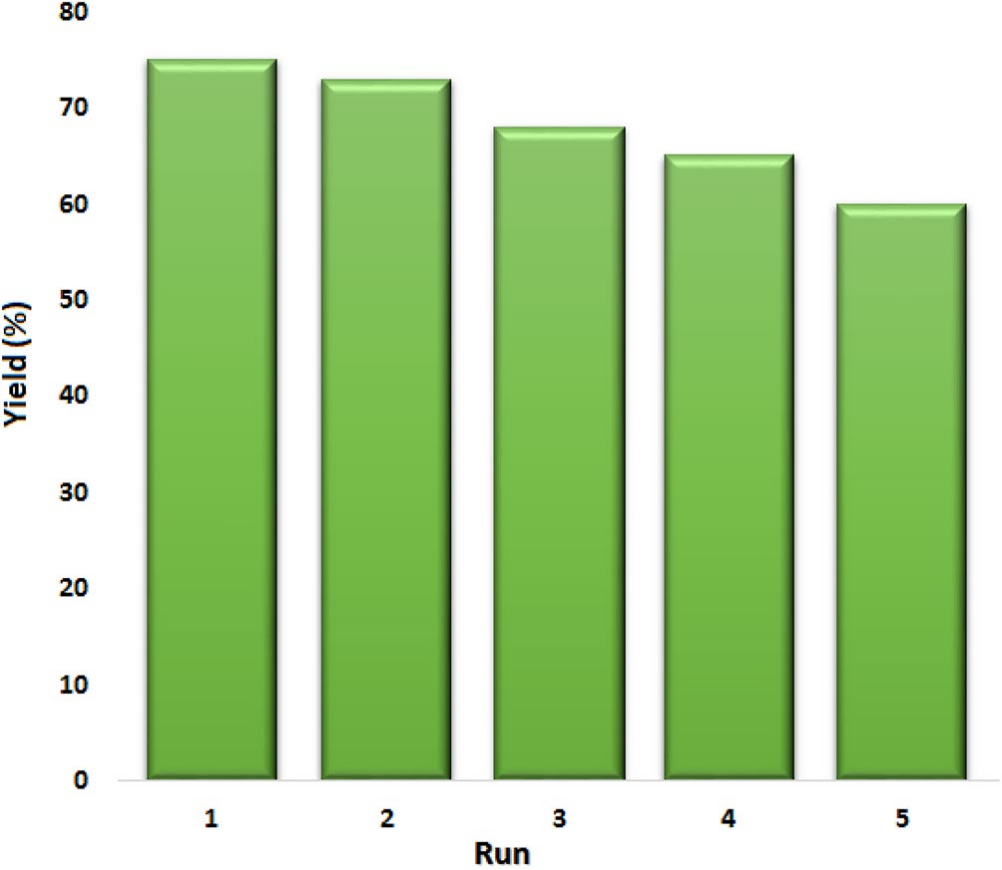
Reusability of N-GOQDs/OH^−^ for the epoxidation of benzalacetophenone.

After completion of the reaction in a homogeneous system, the reaction mixture was diluted with water, and the product was extracted with ethyl acetate. Since the catalyst is completely soluble in water, the catalyst was retained in the aqueous phase. The extracted catalyst in the aqueous layer was concentrated under vacuum to a final volume of 4 mL to reuse in another run. Recycling experiments confirmed the acceptable reusability and chemical stability of the catalyst since the recovered catalyst can be used during four runs (Fig. 6).

Table 3 lists the comparison of catalytic performance of N-GOQDs/OH^−^ as a pseudo-homogeneous catalyst for the epoxidation of benzalacetophenone with various reported catalysts. Compared with some data in the literature, the N-GOQDs/OH^−^ revealed appropriate catalytic performance and good reusability for the epoxidation of benzalacetophenone.

**Table 3:** Catalytic performance of different catalysts for the epoxidation of benzalacetophenone.

Catalyst	Conditions	Yield (%)	Reusability	References
[L-Aaemim]Br	H_2_O_2_, NaOH, DMF, r.t	93	5	^41^
Mg_10_Al_2_(OH)_24_CO_3_	H_2_O_2_, methanol, 40 °C	57	4	^57^
Polyvinylpyrrolidone	ButOOH, NaOH, dioxane, reflux	85	–	^58^
Amberlyst A-26 (OH^−^)	H_2_O_2_, Dioxane, r.t	96	–	^50^
UHP	EtOH/H_2_O, NaOH	91	–	^36^
Amidine	H_2_TPP, O_2_, LEDs, dioxane, 10 °C	93	–	^59^
N-GOQDs/OH^−^	Dioxane/H_2_O, H_2_O_2_, r.t	75	5	

## Conclusion

In summary, quaternary ammonium hydroxide‐functionalized GOQDs has been prepared. The provided GOQDs were characterized using ^1^H NMR, FT-IR, TEM, SEM, XPS, EDX mapping, and fluorescence spectroscopy. On the whole, results of these analysis support the expected structure of N-GOQDs/OH^−^. The catalyst was found to be active towards epoxidation of α,β-unsaturated ketones in the presence of aqueous H_2_O_2_ as a green oxidant, at ambient temperature. The corresponding epoxide products were obtained in good to high yields. Due to the presence of various functional groups on the surface of the N-GOQDs/OH^−^ imparts high solubility in water and polar organic solvents. Therefore substrates and the catalyst can create a homogeneous phase, this helps to achieve a high efficiency of the catalyst. This pseudo-homogeneous catalyst will be widely expected to use for future catalytic applications in oxidation reactions of organic compounds.

## Supplementary Information

Supplementary Information.

## Data Availability

All data generated or analyzed during this study are included in this published article [and its supplementary information files].
