# Supplementary material for: Design and introduction of quaternary ammonium hydroxide‐functionalized graphene oxide quantum dots as a pseudo-homogeneous catalyst for epoxidation of α,β-unsaturated ketones
Source: Sci Rep. 2023 May 19;13:8140. doi: 10.1038/s41598-023-34635-5 (PMC10199001; doi:10.1038/s41598-023-34635-5)

**Supporting Information**

**Design and Introduction of Quaternary Ammonium Hydroxide‐Functionalized Graphene Oxide Quantum Dots as a Pseudo-Homogeneous Catalyst for Epoxidation of α,β-Unsaturated Ketones**

Mohammed Salim Mohammed,^a^ Homa Targhan,^b^ Kiumars Bahrami*^a,b^

^a^Nanoscience and Nanotechnology Research Center (NNRC), Razi University, Kermanshah, 67144-14971, Iran.

^b^Department of Organic Chemistry, Faculty of Chemistry, Razi University, Kermanshah 67144-14971, Iran.

E-mail: [kbahrami2@hotmail.com](mailto:kbahrami2@hotmail.com); [k.bahrami@](mailto:k.bahrami@)razi.ac.ir; Fax: +98(833)4274559; Tel: +98(833)4274559.

**Figure S1.** Schematic procedure for the synthesis of the N-GOQDs/OH catalyst


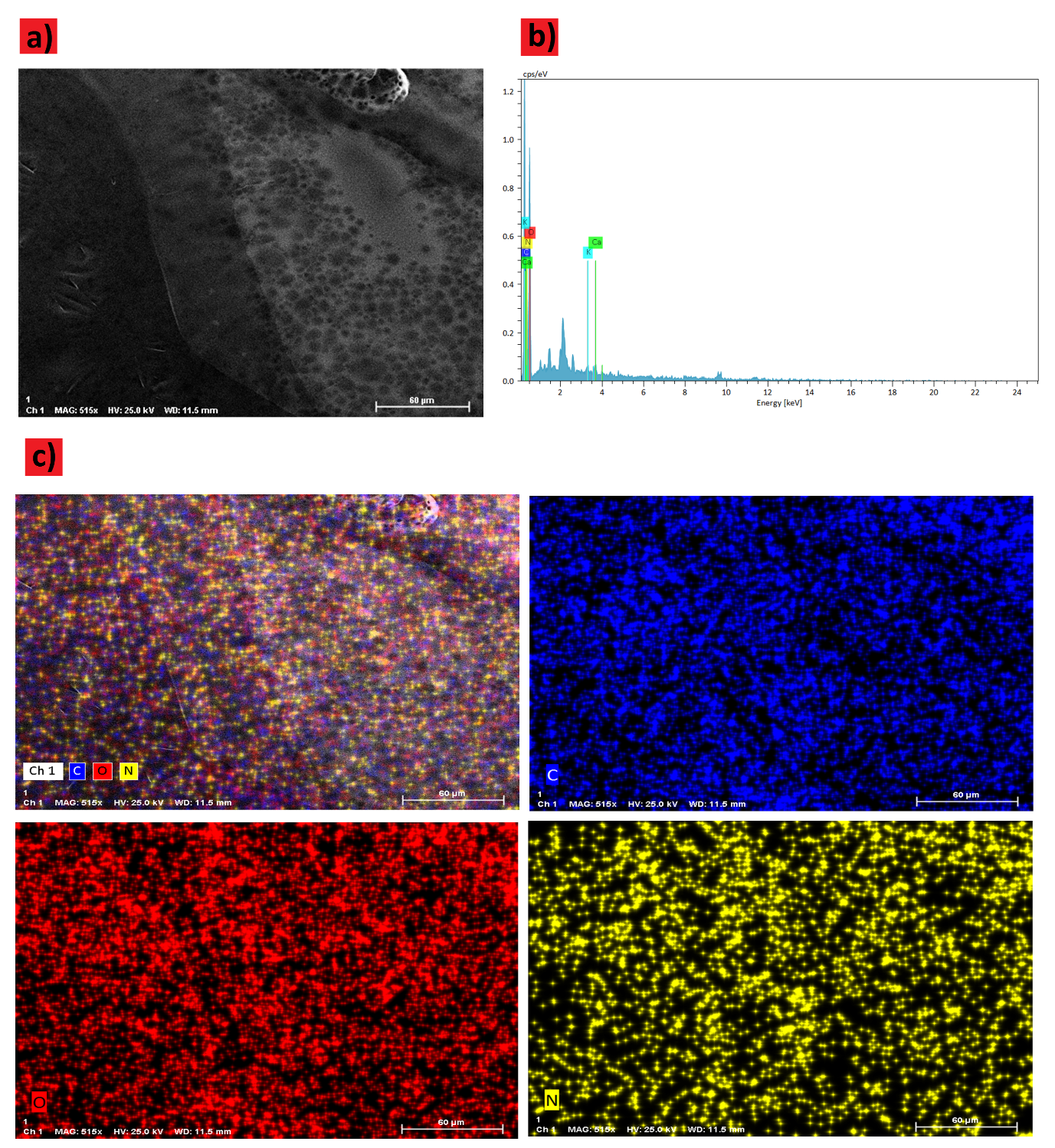


**Figure S2.** The SEM image of the same region as the EDX (a), The EDX mapping of N-GOQDs/OH (b and c).

**Table S1.** The element content table based on EDX data

| Element | Mass Norm. [%] | Atom [%] |
| --- | --- | --- |
| Oxygen | 45.72 | 40.51 |
| Carbon | 28.69 | 33.87 |
| Nitrogen | 25.16 | 25.46 |
| Calcium | 0.28 | 0.10 |
| Potassium | 0.15 | 0.05 |
|  | 100.00 | 100.00 |


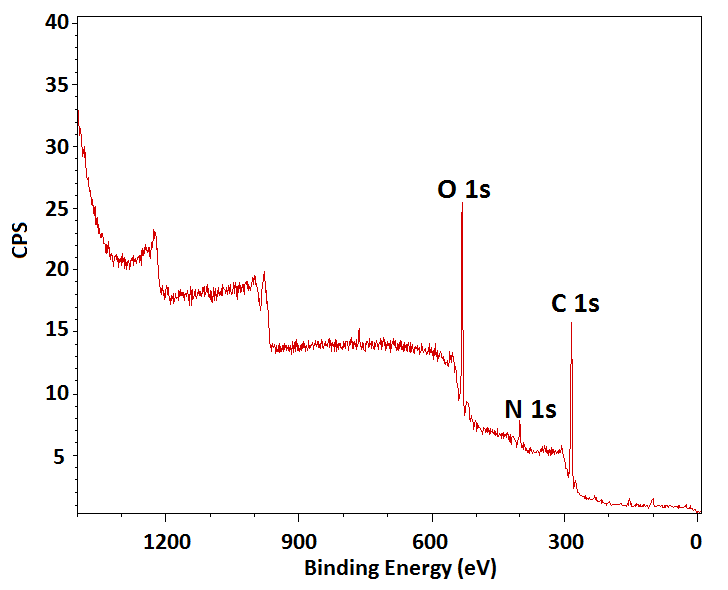


**Figure S3.** XPS spectral of N-GOQDs/OH

**^1^H-NMR and FT-IR data of the selected products**

**Figure S4**. ^1^H-NMR of (4-methoxyphenyl)(3-(4-methoxyphenyl)oxiran-2-yl)methanone (Table 2, Entry 4)


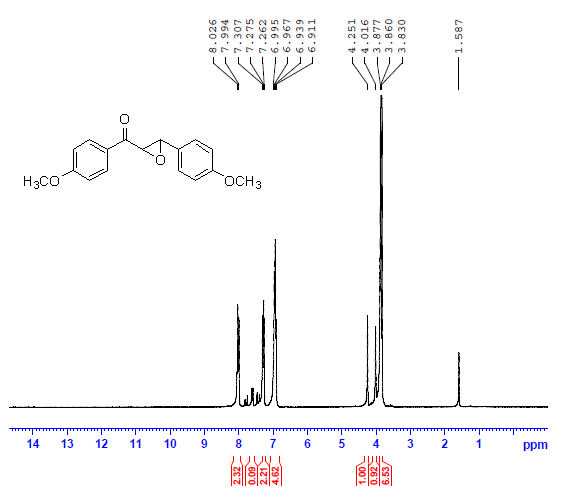


**Figure S5.** ^1^H-NMR of (4-chlorophenyl)(3-(4-chlorophenyl)oxiran-2-yl)methanone (Table 2, Entry 2)


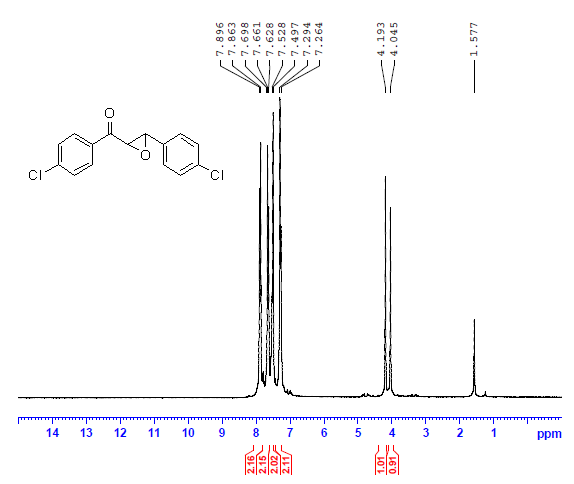


**Figure S6**. FT-IR of (4-methoxyphenyl)(3-(4-methoxyphenyl)oxiran-2-yl)methanone (Table 2, Entry 4)


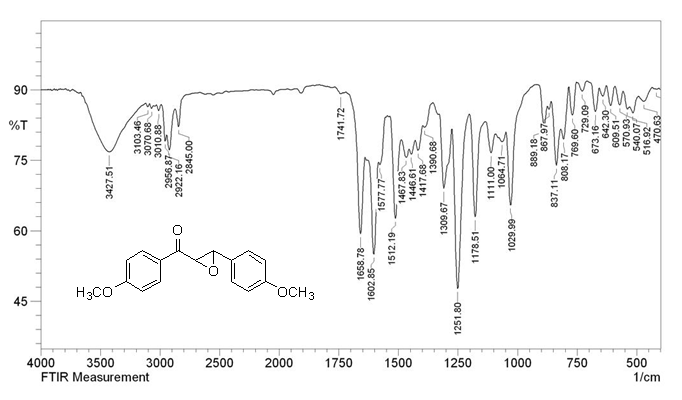


**Figure S7.** FT-IR of (4-chlorophenyl)(3-(4-chlorophenyl)oxiran-2-yl)methanone (Table 2, Entry 2)


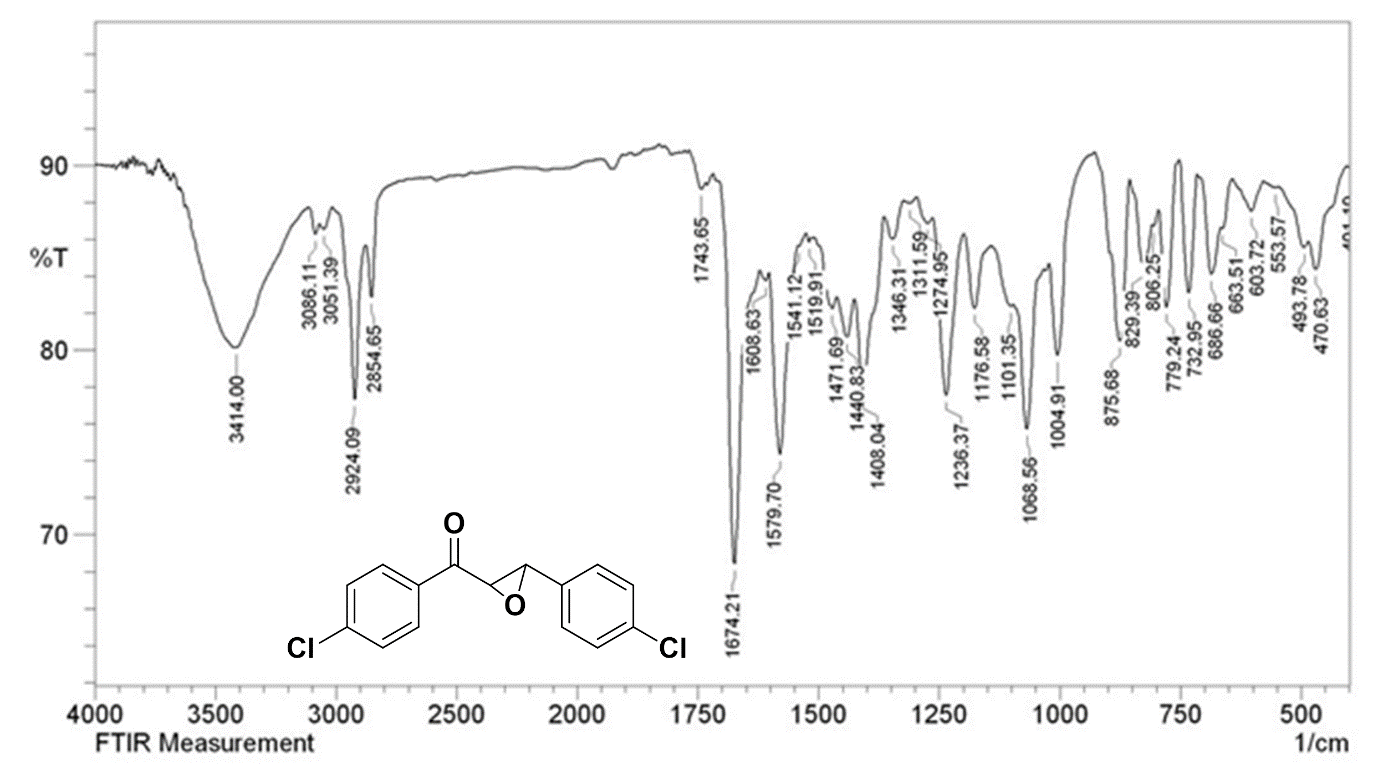

Supplement: Supplementary file 1 — Supplementary Information. [file 41598_2023_34635_MOESM1_ESM.docx]
